# Magnetic Bloch oscillations and domain wall dynamics in a near-Ising ferromagnetic chain

**DOI:** 10.1038/s41467-022-29854-9

**Published:** 2022-05-10

**Authors:** Ursula B. Hansen, Olav F. Syljuåsen, Jens Jensen, Turi K. Schäffer, Christopher R. Andersen, Martin Boehm, Jose A. Rodriguez-Rivera, Niels B. Christensen, Kim Lefmann

**Affiliations:** 1 Niels Bohr Institute, University of Copenhagen, Universitetsparken 5, 2100 Copenhagen, Denmark; 2 Institut Laue-Langevin, CS 20156, 38042 Grenoble Cedex 9, France; 3 Department of Physics, University of Oslo, P. O. Box 1048 Blindern, N-0316 Oslo, Norway; 4 National Centre for Nano Fabrication and Characterization, Technical University of Denmark, 2800 Kgs. Lyngby, Denmark; 5 Institut Laue-Langevin, CS 20156, 38042 Grenoble Cedex 9, France; 6 NIST Center for Neutron Research, National Institute of Standards and Technology, Gaithersburg, MD 20899 USA; 7 Department of Materials Science and Engineering, University of Maryland, College Park, MD 20742 USA; 8 Department of Physics, Technical University of Denmark, 2800 Kgs. Lyngby, Denmark

**Keywords:** Magnetic properties and materials, Ferromagnetism, Quantum mechanics

## Abstract

When charged particles in periodic lattices are subjected to a constant electric field, they respond by oscillating. Here we demonstrate that the magnetic analogue of these Bloch oscillations are realised in a ferromagnetic easy axis chain. In this case, the “particles” undergoing oscillatory motion in the presence of a magnetic field are domain walls. Inelastic neutron scattering reveals three distinct components of the low energy spin-dynamics including a signature Bloch oscillation mode. Using parameter-free theoretical calculations, we are able to account for all features in the excitation spectrum, thus providing detailed insights into the complex dynamics in spin-anisotropic chains.

## Abstract (web summary)

An electron subject to a periodic potential and a constant electric field exhibit oscillatory dynamics, known as Bloch oscillations. Here, the authors demonstrate a magnetic analogue of Bloch oscillations in a ferromagnetic near-Ising chain, where magnetic excitations oscillate in response to a magnetic field.

## Introduction

First described by F. Bloch in 1929^1^, electronic Bloch oscillations (BOs) are the response of charged particles in a periodic potential to a constant electric field^2,3^. The field gives rise to a force *ℏ* *dk*/*dt* = *qE*, which drives the particle through the Brillouin zone. Upon crossing the Brillouin zone boundary the velocity *dE*/*dk* is reversed, leading to oscillatory motion. Observation of Bloch oscillations had to await the development of ultra-pure semiconductor superlattices^4–6^ and ultra-cold atoms in optical potentials^7,8^. BOs have been observed directly in real space in waveguide arrays^9,10^, and more recently in a Bose-Einstein condensate^11^. A magnetic analogue of the electronic BOs was predicted to exist in ferromagnetic near-Ising anisotropic spin-1/2 chains in a magnetic field and CoCl_2_ ⋅ 2H_2_O was identified as a promising candidate material^12,13^. In such chains, an excitation consisting of a domain wall (DW), separating regions of spins pointing up from spins pointing down, can be thought of as an analogue of a charged particle in a periodic potential undergoing BOs in an electric field. The presence of anisotropic couplings is crucial for the magnetic Bloch oscillations (MBOs) in order to create a periodic band for the DW excitation. The magnetic field acts as a force trying to align spins, thus accelerating the DW in one direction, leading to oscillatory motion in the same way as for the charged particle BOs.

For a bulk system, the above picture based on single DW states breaks down due to the large Zeeman energy cost of aligning many spins opposite to the field. Instead, states involving a pair of domain walls (2DW) bounding a short segment of overturned spins have been proposed as more favourable candidates for observation of magnetic Bloch oscillations^14^. In this case the oscillation involves the domain walls at both ends of a small cluster of adjacent spins aligned opposite to the magnetic field direction. In this work, we study the 2DW excitations by inelastic neutron scattering experiments and find evidence for the existence of MBOs in the deuterated, but magnetically identical material CoCl_2_ ⋅  2D_2_O.

## Results

### Spin Hamiltonian

The Hamiltonian of the ferromagnetic near-Ising anisotropic spin-1/2 chain in a longitudinal field is:

1 $${{{{{{{\mathcal{H}}}}}}}}={{{{{{{{\mathcal{H}}}}}}}}}^{I}+{{{{{{{{\mathcal{H}}}}}}}}}^{a}+{{{{{{{{\mathcal{H}}}}}}}}}^{\perp },$$

where

2a $${{{{{{{{\mathcal{H}}}}}}}}}^{I}=-\mathop{\sum}\limits_{i}{{{{{{{{\mathcal{J}}}}}}}}}^{z}{S}_{i}^{z}{S}_{i+1}^{z}-{g}^{z}{\mu }_{B}{\mu }_{0}{H}^{z}\mathop{\sum}\limits_{i}{S}_{i}^{z},$$

2b $${{{{{{{{\mathcal{H}}}}}}}}}^{a}=-\mathop{\sum}\limits_{i}{{{{{{{{\mathcal{J}}}}}}}}}^{a}\left({S}_{i}^{+}{S}_{i+1}^{+}+{S}_{i}^{-}{S}_{i+1}^{-}\right),$$

2c $${{{{{{{{\mathcal{H}}}}}}}}}^{\perp }=-\mathop{\sum}\limits_{i}{{{{{{{{\mathcal{J}}}}}}}}}^{\perp }\left({S}_{i}^{+}{S}_{i+1}^{-}+{S}_{i}^{-}{S}_{i+1}^{+}\right),$$

with ${{{{{{{{\mathcal{J}}}}}}}}}^{z}\, > \,0$, ${{{{{{{{\mathcal{J}}}}}}}}}^{a}=({{{{{{{{\mathcal{J}}}}}}}}}^{x}-{{{{{{{{\mathcal{J}}}}}}}}}^{y})/4$ and ${{{{{{{{\mathcal{J}}}}}}}}}^{\perp }=({{{{{{{{\mathcal{J}}}}}}}}}^{x}+{{{{{{{{\mathcal{J}}}}}}}}}^{y})/4$. The usual spin raising and lowering operators are defined as ${S}_{i}^{\pm }={S}_{i}^{x}\pm i{S}_{i}^{y}$. The total effective *g* factor along the *z*-axis is the sum of the orbital and spin *g* factors: ${g}^{z}={g}_{L}^{z}+{g}_{S}^{z}$.

For an ideal Ising chain (${{{{{{{{\mathcal{J}}}}}}}}}^{a}={{{{{{{{\mathcal{J}}}}}}}}}^{\perp }=0$) in zero magnetic field, the energy levels of all 2DW states, regardless of the number of overturned spins, *l*, are degenerate, since the energy cost of creating a spin cluster comes only from the domain walls at either end of the cluster. In a magnetic field, the energy cost of aligning spins against the field splits the spectrum into a series of equidistant levels. In this case MBOs do not occur: Since $[{{{{{{{\mathcal{H}}}}}}}},{S}_{{{{{{{{\rm{tot}}}}}}}}}^{z}]=0$, the cluster eigenstates all have fixed and time-independent numbers of spins oriented anti-parallel to the field.

MBOs become possible in the presence of anisotropic couplings (${{{{{{{{\mathcal{J}}}}}}}}}^{a}\,\ne \,0$). In this case, too, the spectrum splits into equidistant levels in the presence of a magnetic field, but the cluster wave functions are superpositions of 2DW states involving different numbers of overturned spins. Such a spectrum is known as the magnetic Wannier-Zeeman Ladder (WZL)^13,14^, in full analogy with the electronic Wannier-Stark Ladder^15^, which is the quantum mechanical signature of electronic BOs. When a WZL is formed, the expectation value of the cluster size 〈*l*〉 oscillates with a frequency *ω*_*B*_ corresponding to the energy difference between cluster states that both have contributions from spin clusters of the same size. In the weak-anisotropy limit, this difference is given by the Zeeman energy:

3 $$\hslash {\omega }_{B}={g}^{z}{\mu }_{B}{\mu }_{0}{H}^{z}.$$

The existence of spin cluster excitations in the proposed candidate material CoCl_2_ ⋅ 2H_2_O has already been demonstrated in far-infrared spectroscopy studies, affirming the predominant Ising nature of this system^16,17^.

### The single-chain 2DW approximation

**Fig. 1:**
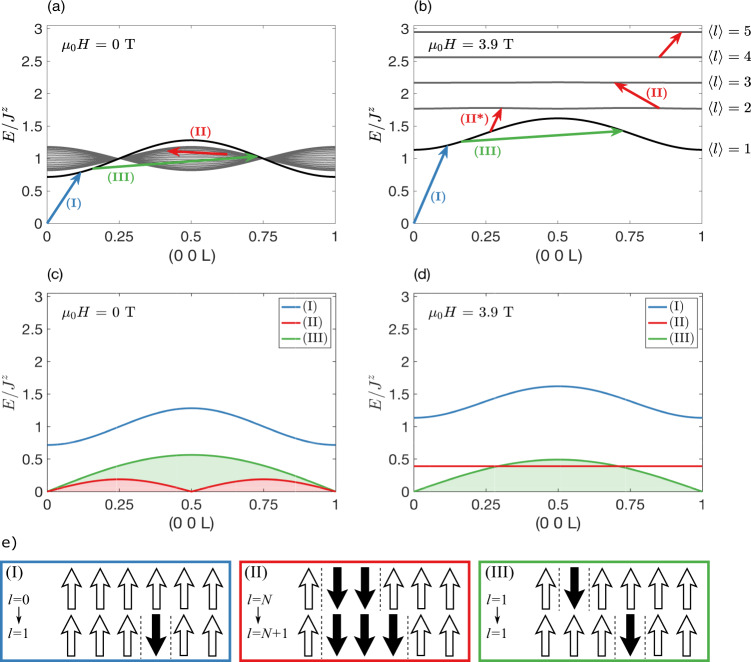
Cluster energy levels and magnetic excitations for a single chain. Using the parameters ${{{{{{{{\mathcal{J}}}}}}}}}^{a}/{{{{{{{{\mathcal{J}}}}}}}}}^{z}=0.05$, ${{{{{{{{\mathcal{J}}}}}}}}}^{\perp }/{{{{{{{{\mathcal{J}}}}}}}}}^{z}=0.12$, ${{{{{{{{\mathcal{J}}}}}}}}}^{z}=3.845$ meV and *g*^*z*^ = 6.602 relevant for a single chain in CoCl_2_ ⋅ 2D_2_O. Panels **a** and **b** show calculated cluster eigenenergies in zero field, *μ*_0_*H*^*z*^ = 0, and in the high field limit where the WZL is well-developed, *μ*_0_*H*^*z*^ = 3.9 T. The energy of the ferromagnetic ground state is set to $E/{{{{{{{{\mathcal{J}}}}}}}}}^{z}=0$. L is the momentum transfer component along the reciprocal lattice vector c^*^ (*Q* = L ⋅ c^*^). **c**, **d** Interactions with neutrons cause three distinct types of transitions between the cluster eigenstates, shown by arrows in **a** and **b** and illustrated in **e**. Each gives rise to a contribution to the dynamic structure factor, shown in **c** and **d**. **e**-(I) Creation of a 〈*l*〉 = 1 (single spin-flip) cluster leads to single-magnon scattering. **e**-(II) Transitions between cluster states characterised by different 〈*l*〉 can move a DW by one site. In zero field, this yields a continuum of periodicity 0.5 r.l.u., while in a magnetic field it causes a well defined MBO mode. The transition from 〈*l*〉 = 1 to 〈*l*〉 = 2 states is a special case and is marked by the symbol (II*). **e**-(III) Intracluster-level transitions for the 〈*l*〉 = 1-cluster are reflected in a nearly field-independent continuum of period 1 r.l.u.

The full energy spectrum of single cluster states (2DW) was calculated by one of us^14^ using parameters corresponding to a single chain in CoCl_2_ ⋅ 2D_2_O. Here we will denote the class of states with mean cluster size 〈*l*〉 by $\left|{\lambda }_{l}\right\rangle$. In zero field the $\left|{\lambda }_{l = 1}\right\rangle$ cluster state lies outside a continuum of $\left|{\lambda }_{l \,{ > }\,1}\right\rangle$ cluster states with smaller bandwidth (Fig. 1a). In a sufficiently large magnetic field, the spin cluster continuum is split and the excitation spectrum now consists of the dispersive $\left|{\lambda }_{l = 1}\right\rangle$ mode and the characteristic WZL of nearly dispersionless modes (Fig. 1b), hence verifying CoCl_2_ ⋅ 2D_2_O as a candidate material for observing MBOs.

The intensity detected in a neutron scattering experiment has contributions from the correlation functions (see Supplementary Note 4)^18^:

4 $${S}^{\alpha \alpha }(Q,\omega )=	 \frac{1}{Z}\mathop{\sum}\limits_{\lambda ,\lambda ^{\prime} }\exp \;(-{E}_{\lambda }/{k}_{B}T)| \langle \lambda ^{\prime} | {S}_{Q}^{\alpha }| \lambda \rangle {| }^{2}\\ 	\times \delta (\hslash \omega -({E}_{\lambda ^{\prime} }-{E}_{\lambda })),$$

where ${S}_{Q}^{\alpha }={\sum }_{i}{e}^{iQ\cdot {R}_{i}}{S}_{i}^{\alpha }$ and *α* = *x*, *y*, or *z*. *Z* is the partition function, while $\left|\lambda \right\rangle$ and $\left|\lambda ^{\prime} \right\rangle$ are states of the sample before and after the scattering event with energies *E*_*λ*_ and ${E}_{\lambda ^{\prime} }$, respectively. Let us now consider how transitions between the cluster energy states discussed above are reflected in three distinct contributions to the neutron scattering intensity.

At *T* = 0, only the ferromagnetic ground state, $\left|{\lambda }_{l = 0}\right\rangle$ with all spins parallel to *H*, is populated. The operators ${S}_{Q}^{x}$ and ${S}_{Q}^{y}$ cause transitions to states with a single spin flipped, as shown in Fig. 1e-(I). The result is a dispersive spin-wave excitation in *S*(*Q*, *ω*) which is found to appear in the range 4–6 meV in zero field^19,20^. The spin-wave contribution exhausts *S*(*Q*, *ω*) at *T* = 0 K, since transition matrix elements between the ground state and higher-lying cluster states (i.e. those dominated by *l* > 1 contributions) are vanishingly small^14^. In particular, it is not possible to probe the WZL, and hence MBOs, at low temperatures.

However, when the temperature is increased, the states of the WZL will be thermally populated. The spectrum is now richer and contains two additional contributions from transitions between different excited 2DW states (Fig. 1e-(II)), and between different momentum states for the same 2DW state (Fig. 1e-(III)). The first of these involves a change in the length of the spin cluster $\left|{\lambda }_{l}\right\rangle \to \left|{\lambda }_{l+1}\right\rangle$ caused by ${S}_{Q}^{x}$ and ${S}_{Q}^{y}$. For sufficiently large fields, this is reflected in *S*(*Q*, *ω*) in the appearance of a low-energy peak at *ℏω* = *ℏω*_*B*_^14^ signifying a WZL spectrum and the existence of MBOs. The special case for the transition $\left|{\lambda }_{1}\right\rangle \to \left|{\lambda }_{2}\right\rangle$, the process-II*, is discussed in the next section. By contrast, in zero field, the process in Fig. 1e-(II) gives rise to a low-energy continuum contribution to the transverse correlation functions *S*^*xx*^(*Q*, *ω*) and *S*^*yy*^(*Q*, *ω*). The upper boundary of this continuum is determined by the bandwidth, $4{{{{{{{{\mathcal{J}}}}}}}}}^{a}$, of the cluster states in Fig. 1a, and can be approximated by:

5 $${E}_{{{{{{{{\rm{II}}}}}}}}}=4{{{{{{{{\mathcal{J}}}}}}}}}^{a}| \sin (2\pi {{{{{{{\rm{L}}}}}}}})| ,$$

where L is the momentum transfer component along with the reciprocal lattice vector c^*^ (*Q* = L ⋅ c^*^). This is the ferromagnetic equivalent of the Villain mode observed in antiferromagnetic Ising chains^21–23^.

**Fig. 2:**
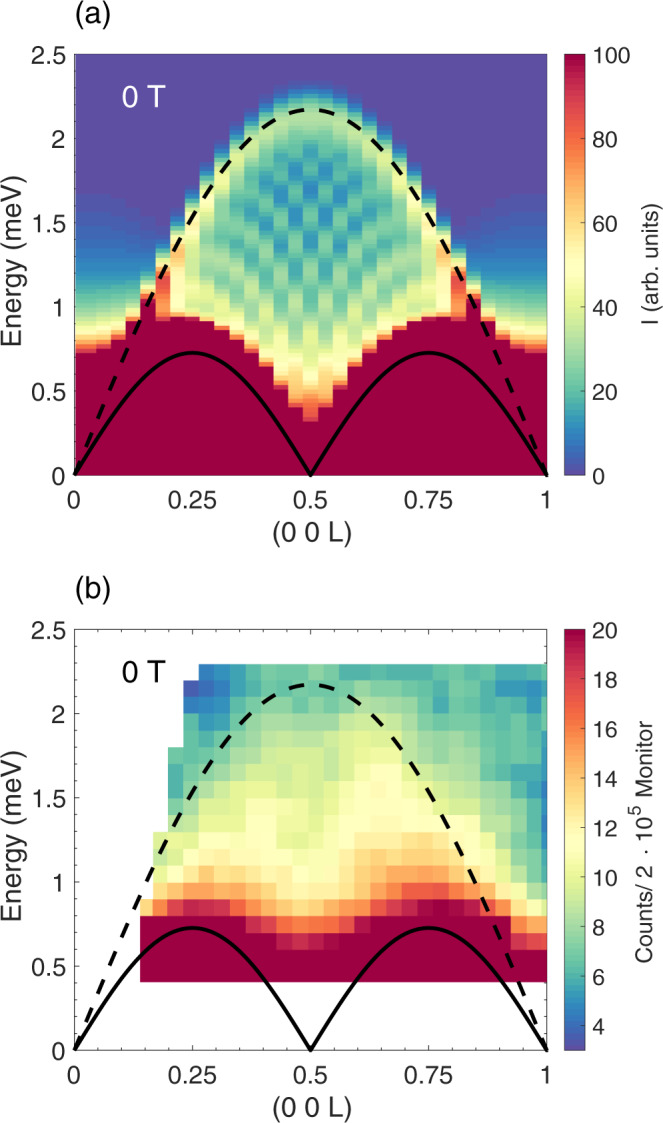
The zero-field scattering continua. **a** Numerical approximation of *S*(*Q*, *ω*) using the same parameters as in Fig. 1. Note that the checkerboard pattern within the continuum is due to finite size effects in the numerical calculation. Equation (5) is superimposed as a black solid line and equation (6) as a black dashed line. **b** Inelastic neutron scattering data at zero field and *T* = 22 K obtained using MACS.

The second finite temperature contribution to *S*(*Q*, *ω*) involves transitions within the first excited 2DW eigenstate, $\left|{\lambda }_{l = 1}\right\rangle \to \left|{\lambda }_{l = 1}\right\rangle$ (Fig. 1e-(III)), and introduces the second continuum in the same low-energy range occupied by the MBO peak in finite field, and by the transverse continuum in zero field. The upper limit of the continuum, which is due to ${S}_{Q}^{zz}$ and hence contributes to the longitudinal correlation function *S*^*zz*^(*Q*, *ω*), is given by:

6 $${E}_{{{{{{{{\rm{III}}}}}}}}}=A| \sin (\pi {{{{{{{\rm{L}}}}}}}})| ,$$

where *A* takes the value $4{{{{{{{{\mathcal{J}}}}}}}}}^{\perp }(1+{({{{{{{{{\mathcal{J}}}}}}}}}^{a}/{{{{{{{{\mathcal{J}}}}}}}}}^{\perp })}^{2})$ in zero magnetic field and approaches $4{{{{{{{{\mathcal{J}}}}}}}}}^{\perp }$ in a strong magnetic field. The two contributions from eq. (5) and (6) are shown in Fig. 2 (See Supplementary Note 6 for derivations of equations (5) and (6)).

### The 3D mean-field RPA model

To get the correct quantitative details of these processes, it is necessary to go beyond the 2DW approximation of the single-chain model in equation (1) to account for collisions between cluster states. It is also crucial to include inter-chain and next-nearest neighbour intra-chain couplings. In the next section, we will therefore compare the in-field inelastic neutron scattering data to numerical calculations based on a mean-field/random phase approximation (RPA). This model considers a short-chain segment of six neighbouring spin-1/2 ions and the spin Hamiltonian established in ref. ^24^. The model accounts for a range of properties of CoCl_2_ ⋅ 2H_2_O and CoCl_2_ ⋅ 2D_2_O including the magnetisation^25^, susceptibility^26^, the spin waves^19,20,27^ and transverse-field quantum criticality^28^ observed by neutron scattering, and the magnetic cluster excitations revealed by far-infrared spectroscopy^17^. Within this model, interactions with neighbouring chains contribute to the effective field experienced by each domain wall. This leads to a modification of the magnetic Bloch oscillation energy, equation (3), which for the case of CoCl_2_ ⋅ 2D_2_O becomes:

7 $$\hslash {\omega }_{B}^{* }={g}^{z}{\mu }_{B}{\mu }_{0}{H}^{z}+\left(4{{{{{{{{\mathcal{J}}}}}}}}}_{1+4}^{z}{{{{{{{{\mathcal{J}}}}}}}}}_{1+2}^{^{\prime} z}{{{{{{{{\mathcal{J}}}}}}}}}_{2}^{z}\right)\langle {S}^{z}\rangle .$$

**Fig. 3:**
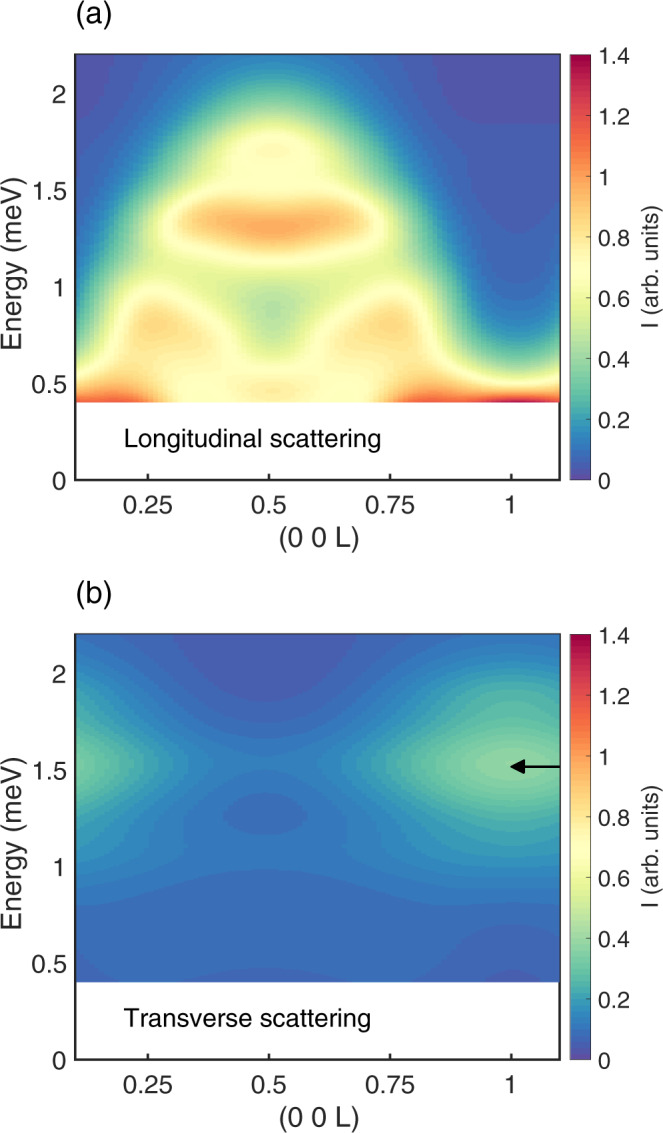
The transverse and longitudinal components of the excitation spectrum. The calculated neutron scattering cross section in Fig. 4d at 22 K and 7 T separated in its **a** longitudinal and **b** transverse parts, proportional to ${({g}^{z})}^{2}{S}^{zz}$ and $0.65{({g}^{x})}^{2}{S}^{xx}+0.35{({g}^{y})}^{2}{S}^{yy}$, respectively (see Supplementary Note 4). The MBO signal is only present in the transverse part of the signal. The black arrows correspond to the prediction of the effective Bloch energy, $\hslash {\omega }_{B}^{* }$, given by equation (7). The colour scale is the same for the two plots.

Here 〈*S*^*z*^〉 is the average polarisation of the spin chains along the field axis. The values of the antiferromagnetic Ising-type inter-chain couplings ${{{{{{{{\mathcal{J}}}}}}}}}_{1}^{z}$, ${{{{{{{{\mathcal{J}}}}}}}}}_{1}^{^{\prime} z}$ and ${{{{{{{{\mathcal{J}}}}}}}}}_{2}^{z}$ were determined in ref. ^24^ and are fixed throughout this paper (more details can be found in Supplementary Note 2). In keeping with our discussions based on Fig. 2 and equation (4), the RPA model predicts that the magnetic Bloch oscillation mode is transverse, while the type-III continuum scattering is longitudinal. This is illustrated in Fig. 3. In addition the calculations highlight that although the MBOs corresponds to transitions between different levels of the WZL, which are approximately non-dispersive in the high field limit, the neutron scattering intensity nevertheless depends on the component of the neutron momentum transfer along c^*^ with clear maxima at integer values of L. Finally, we discuss the signature of processes-II^*^ connecting dispersive $\left|{\lambda }_{l = 1}\right\rangle$ to nearly dispersionless $\left|{\lambda }_{l = 2}\right\rangle$ domain wall states (see Fig. 1b). In the 2DW approximation, these cause an intensity maximum $2{{{{{{{{\mathcal{J}}}}}}}}}_{\perp }$ above the intensity maximum of type-II processes at the zone-boundary L = 1 r.l.u. A more accurate estimate is obtained by the cluster RPA method, which is found to be in perfect agreement with exact diagonalisation calculations that include states beyond the 2DW approximation, when addressing the same model. The RPA then shows, that the negative intra-chain next-nearest-neighbour interaction, appearing in the more realistic model, reduces the difference between the intensity maxima due to processes of type-II* and type-II. The difference is reduced from 2*J*⊥ ≃ 0.9 meV to about 0.25 meV. In the experimental data described below, the two contributions form a combined broadened MBO signal.

### Neutron scattering

We now turn to present our experimental results obtained using the neutron spectrometers MACS^29^ and ThALES^30,31^. The MACS spectrometer is optimised for the study of weak and diffuse contributions to *S*(*Q*, *ω*) covering an extended *Q*-range. In the instrument configuration chosen the maximum energy transfer was constrained to 2.2 meV, which in turn limited the search for MBO signatures to fields *μ*_0_*H* ≤ 9 T. Additional measurements were carried out at the ThALES spectrometer, focusing on a single reciprocal lattice point, but using a set-up that allowed to track the MBO signal at higher energy transfers and magnetic fields. The chosen setups (see Methods) improve on two previous unsuccessful attempts at identifying MBOs in CoCl_2_ ⋅ 2D_2_O^20,27^, by having a higher flux, better energy and momentum transfer coverage and better energy resolution. The experiments were carried out at 22 K, which is slightly above the Néel temperature *T*_*N*_ = 17.2 K^19^. At this temperature, we expect the system to be well-approximated as 1D chains, while ensuring a significant thermal population of the excited 2DW states involved in the MBOs.

The single-chain 2DW calculations in Fig. 2a can be seen to reproduce the salient features of the experimental data: There is a period 1 r.l.u. continuum peaked at $\left(0\,0\,\frac{1}{2}\right)$ and bounded by equation (6) with a realistic pre-factor of *A* = 2.17 meV, given by the coupling constants for a single chain in CoCl_2_ ⋅ 2D_2_O. At lower energies we observe a period-0.5 r.l.u. continuum peaked at $\left(0\,0\,\frac{1}{4}\right)$ and $\left(0\,0\,\frac{3}{4}\right)$, and bounded by a curve similar to equation (5), but with a pre-factor slightly exceeding the expectation.

**Fig. 4:**
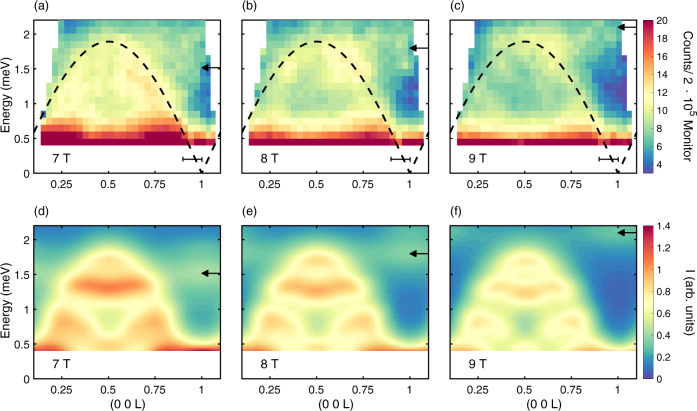
Inelastic neutron scattering data compared to RPA calculations. **a**–**c** Experimental data collected at 22 K at MACS in magnetic fields 7 T, 8 T and 9 T, respectively. **d**–**f** The calculated inelastic neutron scattering cross section, leaving out the variation due to the magnetic form factor (see Supplementary Note 2), as determined by the six spin-1/2 cluster model^24^. The black arrows correspond to the prediction of the effective Bloch energy, $\hslash {\omega }_{B}^{* }$, given by equation (7). The black bars in **a**–**c** represent the data integration range used to produce the data in Fig. 5.

Figure 4a-c show neutron scattering intensity maps as a function of energy and momentum transfer measured at 22 K for magnetic fields of 7 T, 8 T and 9 T, respectively. For comparison, Fig. 4d–f show the corresponding calculated excitation spectra. For all three magnetic fields the overall agreement between data and calculations is clear. The spectra are dominated by a largely field-independent type-III continuum that peaks at $Q=\left(0\,0\,\frac{1}{2}\right)$ originating from the scattering between the thermally populated $\left|{\lambda }_{l = 1}\right\rangle$ states, bounded by equation (6). Outside the boundaries of this continuum, and close to (0 0 1), it is possible to discern a weaker and clearly field-dependent contribution to *S*(*Q*, *ω*), consistent with the expectations for magnetic Bloch oscillations, illustrated by Figs. 1d and 3b. The model calculations, Figs. 4d–f predict a weak mode moving to higher energies with increasing field strength. The same phenomenon can be seen in the corresponding experimental data. It is worth noting that as the field increases, the period-0.5 r.l.u. continuum, equation (5), is seen to lose intensity in both data and model calculations.

**Fig. 5:**
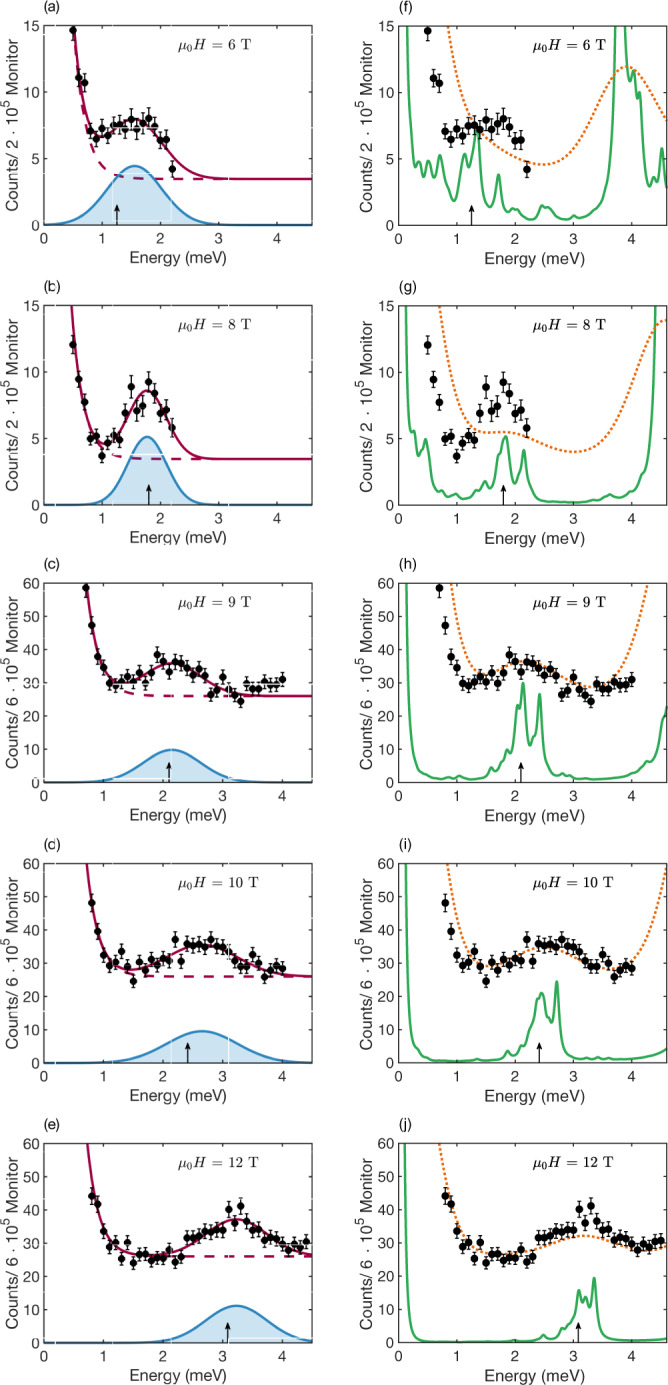
Inelastic Neutron Scattering excitation spectra. Neutron scattering intensity for magnetic fields of 6 T, 8 T, 9 T, 10 T and 12 T. Panels **a**–**e** show excitation spectra together with the fit to the data (solid red lines) and background (dashed red lines) described in the main text and the shaded blue area to their difference—the MBO signal. Black arrows are the predicted effective Bloch energies, $\hslash {\omega }_{B}^{* }$, given by equation (7). Panels **f**–**j** show excitation spectra together with the RPA calculations of the spectra (solid green line) and the RPA calculations with a Gaussian convolution (dashed orange line) as described in the main text. **a**, **b**, **f**–**g** Data measured at MACS, averaged over the momentum range *Q*_L_ = 0.9–1.0 r.l.u. at magnetic fields of 6 T and 8 T. **c**–**e**, **h**–**j** Data measured at ThALES at *Q*_L_ = 1.0 r.l.u^31^ at magnetic fields of 9 T, 10 T and 12 T. Please note the different energy transfer range of the two experiments (see Methods). Error bars represent one standard deviation.

Figure 5 illustrates the evolution with the magnetic field of the observed neutron intensity, for selected values of the magnetic field (Additional raw data are shown in Supplementary Note 8). The signal that we attribute to the MBOs is clearly peaked and increases in energy with increasing field, while its amplitude remains approximately constant. In order to isolate the signal originating from MBOs, we subtract an effective background model reflecting incoherent scattering and a field-dependent contribution from the edge of type-III continuum. The corresponding calculated green curves in Fig. 5f–j show the two peaks originating from the MBO process and the $\left|{\lambda }_{l = 1}\right\rangle \to \left|{\lambda }_{l = 2}\right\rangle$ process. Also shown are the calculated curves convoluted with a Gaussian function (dashed orange line) in order to account for the instrument resolution and the significant thermal broadening at 22 K. An unambiguous determination of the optimal Gaussian width was not possible, but by using FWHM = 1.1 meV, the mean width of the fits to the data matches the mean width of the broad peak around the $\hslash {\omega }_{B}^{* }$ position at in the convoluted RPA calculations, as shown in Fig. 5. Both in the convoluted RPA calculations and in the data, it is no longer possible to separate the contribution from the type-II MBO signal and the type-II* $\left|{\lambda }_{l = 1}\right\rangle \to \left|{\lambda }_{l = 2}\right\rangle$ process.

**Fig. 6:**
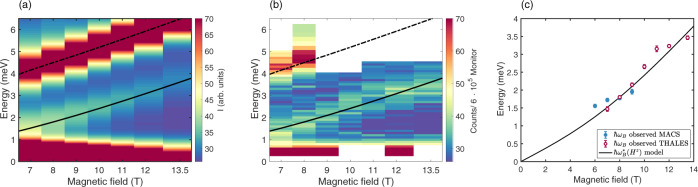
Field dependence of the Bloch energy. The field dependence of the RPA calculations at *Q*_L_ = 1.0 r.l.u are shown in panel **a** convoluted with a Gaussian function as described in the main text. The corresponding data measured at ThALES are shown in panel **b**. The positions of the MBO peak for the energy scans shown in Fig. 5 and in Supplementary Figs. 8 and 9 are shown in panel **c**. The error bars correspond to one standard deviation on the parameter in the fit to the experimental data. Results obtained from MACS data are shown with solid blue circles and results obtained from ThALES data are shown with open red circles. In all panels, the black lines correspond to the predicted Bloch energy $\hslash {\omega }_{B}^{* }$, equation (7) which contains no free parameters. In panels **a**, **b**, the black dashed lines correspond to the position of spin-wave excitation, between $\left|{\lambda }_{l = 0}\right\rangle \to \left|{\lambda }_{l = 1}\right\rangle$ spin cluster states.

In Figure 6 we present the values of the Bloch energy obtained from the Gaussian fits in Fig. 5 along with the predicted effective Bloch energy, equation (7). The spin polarisation, 〈*S*^*z*^〉, was calculated using the RPA model, which has no free parameters.

## Discussion

We see good agreement at all field values above 7 T, but a discrepancy between data and model for the two lowest fields. Decreasing the field leads to a lower polarisation of the spin chains, which in turn means that the spins will be exposed to a broader distribution of effective fields arising from the coupling to neighbouring chains. This will result in a broader distribution of Bloch energies. In addition, at lower magnetic fields, there will be a larger background contribution from the continuum which is difficult to separate from the signal coming from the MBOs. As a result, the systematic uncertainty on the Bloch frequency at 6 and 7 T, in reality will be larger than the standard deviation uncertainty from the fits shown as error bars in Fig. 6. Resolving this discrepancy will require more detailed theoretical modelling and is beyond the scope of this work.

In conclusion, we have studied the field dependence of the low-energy excitation spectrum in CoCl_2_ ⋅ 2D_2_O at 22 K. We observed two thermally induced excitation continua that can be described using the anisotropic and perpendicular parts of the exchange couplings, ${{{{{{{{\mathcal{J}}}}}}}}}^{a}$ and ${{{{{{{{\mathcal{J}}}}}}}}}^{\perp }$. In addition we have identified a broad peak close to the zone boundary which moves to higher energies with increasing field, consistent with the expected signature of magnetic Bloch oscillations and the $\left|{\lambda }_{l = 1}\right\rangle \to \left|{\lambda }_{l = 2}\right\rangle$ cluster transition. Our parameter-free RPA model calculations reproduce all three scattering contributions and in particular provide an overall good quantitative agreement with the field dependence of the zone-boundary mode. We therefore conclude that we have established the existence of MBOs.

Our results provide insights into the domain wall dynamics of anisotropic spin chains and add magnetic Bloch oscillations^13^ to the list of phenomena (including the spin Peierls transition^32^, Dirac magnons^33^ and spinon Fermi surfaces^34^) initially introduced in the study of electrons in periodic solids, and subsequently observed to exist in model quantum magnets as well.

## Methods

### Sample preparation

Two single crystals of CoCl_2_ ⋅ 2D_2_O with a total mass of 1.7 g were grown from a D_2_O-solution by slow evaporation at 70 °C. Their crystal structure was checked using X-ray diffraction and was found to be consistent with literature values: *a* = 7.256 Å, *b* = 8.575 Å, *c* = 3.554 Å and *β* = 97. 6°, in the monoclinic space group C2/m (*#*12)^35^. Deuterated crystal water is used in order to minimise the incoherent neutron background that would otherwise be significant in the case of CoCl_2_ ⋅ 2H_2_O.

### Measurements

The inelastic neutron scattering measurements were carried out at the MACS spectrometer at the NIST Center for Neutron Research^29^ and the ThALES spectrometer at the Institut Laue-Langevin^30^. For all experiments two single crystals of CoCl_2_ ⋅ 2D_2_O were co-aligned in the (H,0,L) plane on an aluminium holder in a vertical field cryomagnet allowing for magnetic fields along the easy-axis (0,K,0)-direction. The area of the two co-aligned samples was ~22 mm wide and 17 mm high.

The measurements at MACS were performed with a fixed final energy of *E*_*f*_ = 3.0 meV. A Be-filter was placed before the monochromator and a BeO-filter after the sample. This configuration is optimal for studying weak signals but limits the neutron energy transfer to 2.2 meV. The scattered beam was analysed by a 20 channel detection system equipped with double-bounce pyrolytic graphite analyser crystals^29^. The sample orientation with respect to the incident beam was fixed in all measurements. For each energy transfer, *S*(*Q*, *ω*) was probed for several orientations of the detection system. Measurements were done in magnetic fields of 0, 6, 7, 8 and 9 T in a vertical field cryomagnet. The beam size at the sample position at MACS was defined by a diaphragm in the incoming beam, with a horizontal slit opening of 44 mm and a vertical slit opening of 90 mm.

Another set of measurements were carried out at the cold triple-axis spectrometer ThALES. Data were collected with a fixed final energy of 4.98 meV (*k*_*f*_ = 1.55 Å^−1^) using pyrolytic graphite monochromator and analyser. A velocity selector was used to filter out higher order neutrons in the incoming neutron beam and a Be-filter was placed in between the sample and the analyser. Measurements were done in magnetic fields of 0, 7, 8, 9, 10, 11, 12 and 13.5 T in a vertical field cryomagnet. The beam size at the sample position at ThALES was defined by a diaphragm in the incoming beam, with a horizontal slit opening of 20 mm and a vertical slit opening of 30 mm.

## Supplementary information

Supplementary Information

## Data Availability

All relevant data are available from the corresponding author.

## Code availability

Codes used for this study are available upon request by contacting the corresponding author.
