# Supplementary material for: Magnetic Bloch oscillations and domain wall dynamics in a near-Ising ferromagnetic chain
Source: Nat Commun. 2022 May 10;13:2547. doi: 10.1038/s41467-022-29854-9 (PMC9090816; doi:10.1038/s41467-022-29854-9)
Supplement: Supplementary file 1 — Supplementary Information [file 41467_2022_29854_MOESM1_ESM.pdf]

Supplementary Information: Magnetic Bloch Oscillations and domain wall dynamics in a near-Ising ferromagnetic chain

Ursula B. Hansen*,[1,2] Olav F. Syljuåsen,[3] Jens Jensen,[1] Turi K. Schäffer,[1] Christopher R. Andersen,[1,4] Martin Boehm,[2] Jose A. Rodriguez-Rivera,[5,6] Niels B. Christensen,[7] and Kim Lefmann[1]

[1]*Niels Bohr Institute, University of Copenhagen, Universitetsparken 5, 2100 Copenhagen, Denmark*
[2]*Institut Laue-Langevin, CS 20156, 38042 Grenoble Cedex 9, France*
[3]*Department of Physics, University of Oslo, P. O. Box 1048 Blindern, N-0316 Oslo, Norway*
[4]*National Centre for Nano Fabrication and Characterization, Technical University of Denmark, 2800 Kgs. Lyngby, Denmark*
[5]*NIST Center for Neutron Research, National Institute of Standards and Technology, Gaithersburg, Maryland 20899, USA*
[6]*Department of Materials Science and Engineering, University of Maryland, College Park, MD 20742, USA*
[7]*Department of Physics, Technical University of Denmark, 2800 Kgs. Lyngby, Denmark*

SUPPLEMENTARY NOTE 1: CRYSTAL STRUCTURE

$CoCl_2 \cdot 2\,H_2O$ has a monoclinic unit cell and belongs to the space group C2/m (#12) with lattice parameters $a = 7.256$ Å, $b = 8.575$ Å, $c = 3.554$ Å and $\beta = 97.6°$ [1]. There are two Co^{++} sites per unit cell: at $(0, 0, 0)$ and $(\frac{1}{2}, \frac{1}{2}, 0)$, with identical surroundings and the Co-ions form chains along the c-axis. The crystal structure of $CoCl_2 \cdot 2\,H_2O$ is shown in Supplementary Figure 1. The single crystals of $CoCl_2 \cdot 2\,D_2O$ were prepared at the Institute of Chemistry at the University of Copenhagen and were grown from a D_2O-solution by slow evaporation at 70 °C. Deuterated crystal water is used in order to minimise the incoherent neutron background that would otherwise be significant in the case of $CoCl_2 \cdot 2\,H_2O$. The two systems have the same symmetry group and similar lattice parameters [2].

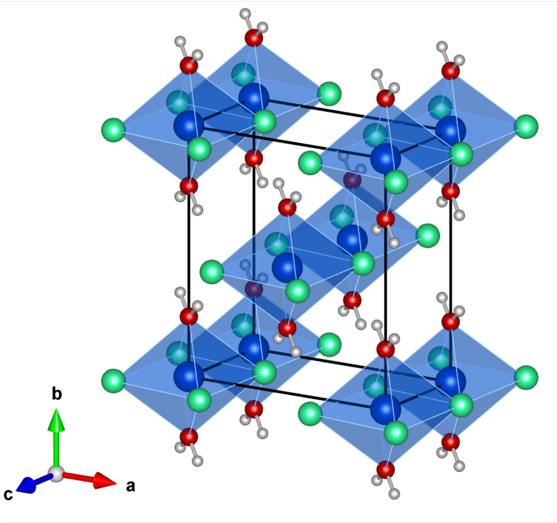

FIG. 1. The monoclinic crystal structure of $CoCl_2 \cdot 2\,H_2O$ (# 12). The colour code of the elements: Cobalt atoms are blue, Chloride atoms are green, Oxygen atoms are red and Hydrogen atoms are grey. The crystal structure drawings was generated using the VESTA program [13].

SUPPLEMENTARY NOTE 2: MAGNETIC EXCHANGE CONSTANTS IN $COCL_2 \cdot 2\,D_2O$

The ground state of the free Co^{2+}-ion is ^4F ($L = 3$ and $S = 3/2$), derived from the electronic configuration $3d^7$ [3]. However, every Co^{2+} sits in a distorted octahedral coordination of four chloride and two oxygen atoms and the strong crystal field combined with the spin orbit coupling splits the ground state into six Kramers doublets. The energy difference between the two lowest lying doublets is 18.8 meV and the following excited doublet lies around 100 meV above the ground state [3, 4]. Neglecting all other states than the ground state doublet of the Co^{2+} ions, the magnetic properties of $CoCl_2 \cdot 2\,D_2O$ can be described within an effective $S = 1/2$ Ising-like anisotropic exchange model [5, 6].

The dominant interactions in $CoCl_2 \cdot 2\,D_2O$ are ferromagnetic and couple nearest neighbour spins lying on chains along the c-axis. Weaker inter-chain interactions give rise to an antiferromagnetic ordering of the chains below $T_N = 17.2$ K. The easy axis corresponds to the crystallographic b-axis. The main exchange couplings of $CoCl_2 \cdot 2\,D_2O$ were earlier determined by some of us [4] using a mean field/random phase approximation. The strength of the isotropic exchange couplings within the $S = 3/2$ model are listed in Supplementary Table I and are shown together with the crystal lattice in Supplementary Figure 2.

TABLE I. The isotropic exchange parameters of $CoCl_2 \cdot 2\,D_2O$ in the $L = 3$, $S = 3/2$ basis and in units of meV [4].

$J(i,j)$	J_0	J_0'	J_1	J_1'	J_2
$\vec{r}_i - \vec{r}_j$	$\pm\vec{c}$	$\pm 2\vec{c}$	$\pm\frac{1}{2}\vec{a} \pm \frac{1}{2}\vec{b}$	$\pm(\frac{1}{2}\vec{a} + \vec{c}) \pm \frac{1}{2}\vec{b}$	$\pm\vec{a}$
(meV)	0.66	-0.08	-0.045	-0.085	-0.025

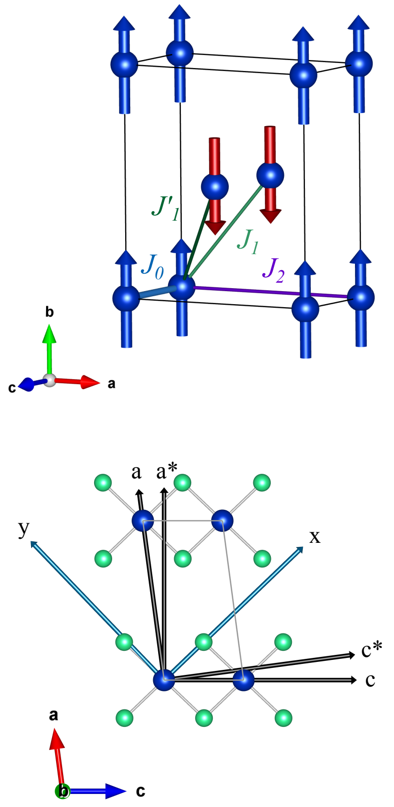

FIG. 2. (top) The main exchange paths in $CoCl_2 \cdot 2\,D_2O$, with the strongest interactions being the coupling J_0, giving rise to ferromagnetic coupled chains running along the c axis. The blue and red arrows represent the antiferromagnetic structure. (bottom) The coordinate system used in this work, showing the x and y directions which corresponds to the Co−Cl bond directions, and the easy and hard axes of magnetisation, respectively [7]. The crystal structure drawings was generated using the VESTA program [13].

The isotropic $S = 3/2$ exchange couplings $J_{i,j}$ listed in Supplementary Table I can be related to the effective $S = 1/2$ model with anisotropic exchange couplings by the expression:

$$\mathcal{J}_{i,j}^{\zeta} = \left(\frac{g_S^{\zeta}}{2}\right)^2 J_{i,j} \qquad (1)$$

where $\zeta = x$, y or z and the total effective g^{ζ}-factor along the ζ-axis is the sum of the orbital and spin g-factors: $g^{\zeta} = g_L^{\zeta} + g_S^{\zeta}$, that are cited in Supplementary Table II. The resulting exchange couplings in the $S = 1/2$ are given in Supplementary Table III. The anisotropy of the Hamiltonian is characterised by the parameters:

$$\mathcal{J}_{i,j}^{a} = \frac{\mathcal{J}_{i,j}^{x} - \mathcal{J}_{i,j}^{y}}{4} \qquad (2)$$

$$\mathcal{J}_{i,j}^{\perp} = \frac{\mathcal{J}_{i,j}^{x} + \mathcal{J}_{i,j}^{y}}{4}. \qquad (3)$$

TABLE II. The effective g factors [4].

	x	y	z
g_L	0.692	0.062	1.775
g_S	2.817	1.879	4.827
g	3.509	1.941	6.602

TABLE III. The interchain exchange parameters of $CoCl_2 \cdot 2\,D_2O$ in the $S = 1/2$ basis and in units of meV [4].

ζ	x	y	z	a	$\perp$
$\mathcal{J}_0^{\zeta}$ (meV)	1.309	0.583	3.844	0.182	0.473
$\mathcal{J}_0^{\prime\zeta}$ (meV)	-0.159	-0.071	-0.466	-0.022	-0.057
$\mathcal{J}_1^{\zeta}$ (meV)	-0.089	-0.040	-0.262	-0.012	-0.032
$\mathcal{J}_1^{\prime\zeta}$ (meV)	-0.169	-0.075	-0.495	-0.023	-0.061
$\mathcal{J}_2^{\zeta}$ (meV)	-0.050	-0.022	-0.146	-0.007	-0.018

SUPPLEMENTARY NOTE 3: CALCULATION OF $\langle S^z \rangle$

The polarisation of the chains, $\langle S^z \rangle$, is field dependent and even at 9 T we do not have a complete polarisation of the chains at 22 K. Using the before mentioned RPA/MF method we have calculated $\langle S^z \rangle$ shown in Supplementary Figure 3 as a function of applied magnetic field. The magnetic field experienced at each chain when an external magnetic field H^z is applied is given by:

$$h_{\text{eff}} = g^z \mu_B \mu_0 H^z + (4J_1 + 4J_1' + 2J_2)(g_S^z/2)^2 \langle S^z \rangle. \quad (4)$$

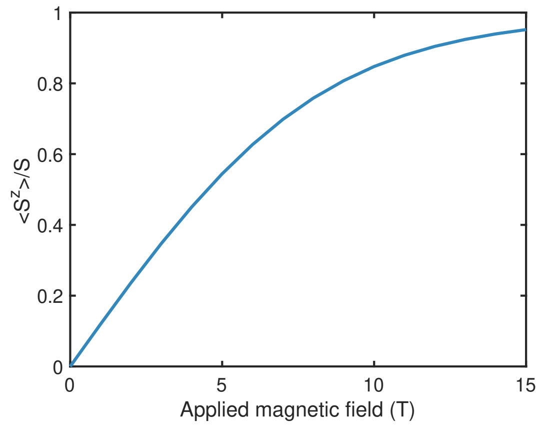

FIG. 3. $\langle S^z \rangle / S$ as a function of applied field calculated at 22 K for the $S = 1/2$ model using 6 spins per cluster.

SUPPLEMENTARY NOTE 4: INELASTIC NEUTRON SCATTERING CROSS SECTION

In a neutron scattering experiment, the number of neutrons detected in the solid angle $d\Omega$ and energy interval dE_f is proportional to the double differential scattering cross section:

$$\frac{d^2\sigma}{d\Omega dE_f} = \frac{k_f}{k_i}(\gamma r_0)^2 e^{-2W(Q)}|\frac{1}{2}f(Q)|^2 S(\mathbf{Q},\omega) \quad (5)$$

$$S(\mathbf{Q},\omega) = \sum_\alpha (g^\alpha)^2 \left(1 - \frac{Q_\alpha^2}{Q^2}\right) S^{\alpha\alpha}(\mathbf{Q},\omega). \quad (6)$$

Here k_i (k_f) is the neutron initial (final) wave vector, γ is the gyromagnetic ratio of the neutron and $r_0 = e^2/mc^2$ is the classical electron radius, $e^{-2W(Q)}$ is the Debye-Waller factor and $f(Q)$ the magnetic form factor. For small values of Q, the magnetic form factor is close to unity and falls of smoothly for large values of Q [8]. In all our calculations $f(Q)$ has been set to 1.

For the coordinate system depicted in Supplementary Figure 2 the longitudinal and transverse contributions to the dynamical correlation function at $\vec{Q} = (0, 0, Q_\mathrm{L})$ can be written as:

$$S^{||}(Q_\mathrm{L},\omega) = (g^z)^2 S^{zz}(Q_\mathrm{L},\omega), \quad (7)$$

$$S^{\perp}(Q_\mathrm{L},\omega) = 0.65(g^x)^2 S^{xx}(Q_\mathrm{L},\omega) + 0.35(g^y)^2 S^{yy}(Q_\mathrm{L},\omega). \quad (8)$$

SUPPLEMENTARY NOTE 5: ADDITIONAL ENERGY LEVEL DIAGRAMS FOR INTERMEDIATE MAGNETIC FIELDS

In Supplementary Figure 4 we show energy level diagrams for field values in between $\mu_0 H = 0$ T and $\mu_0 H = 3.9$ T, which have been shown in Figure 1 of the main text. These calculations are as well made for a single chain of $\mathrm{CoCl_2 \cdot 2\,D_2O}$ [9], which implies that in the presence of inter-chain couplings the value of the external magnetic field should be adjusted according to Supplementary Eq. 4.

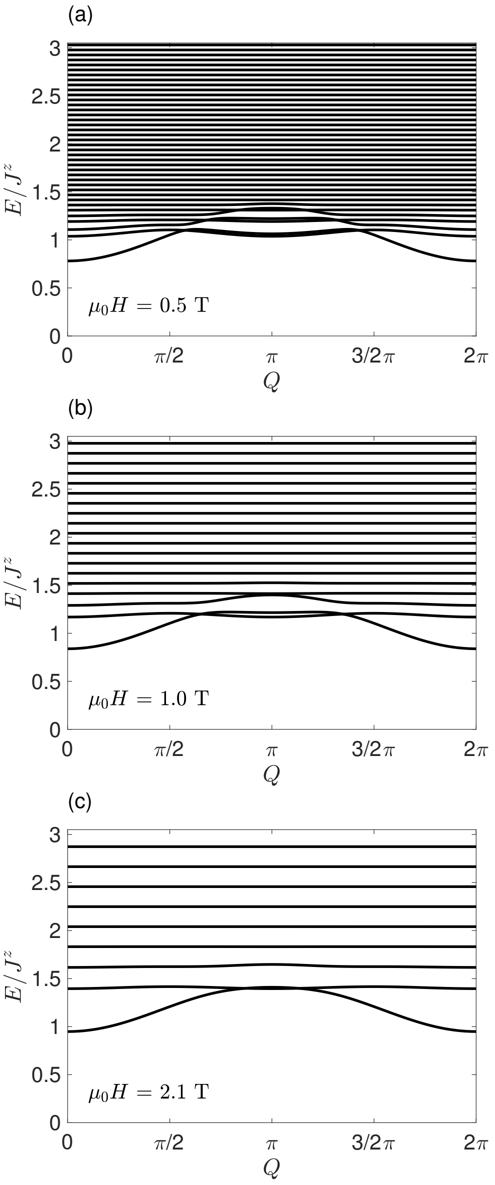

FIG. 4. Additional cluster energy level diagrams. Calculated cluster eigenenergies for (a) $\mu_0 H = 0.5$ T, (b) $\mu_0 H = 1.0$ T and (c) $\mu_0 H = 2.1$ T. The parameters used are the same as in Figure 1 of the main text: $\mathcal{J}^a/\mathcal{J}^z = 0.05$, $\mathcal{J}^\perp/\mathcal{J}^z = 0.12$, $\mathcal{J}^z = 3.845$ meV and $g^z = 6.602$ relevant for a single chain in $\mathrm{CoCl_2 \cdot 2\,D_2O}$.

SUPPLEMENTARY NOTE 6: THE ZERO-FIELD SCATTERING IN $\mathrm{COCL_2 \cdot 2\,D_2O}$

In zero field, there are two types of low energy excitations from the FM ground state: a) spin waves are single spin flips generated by $\mathcal{H}_\perp$, and have a dispersion $E_{\mathrm{sw},p} = \mathcal{J}^z - 2\mathcal{J}^s\cos(p)$; where $\mathcal{J}^s \equiv \mathcal{J}^\perp\left(1 + (\mathcal{J}^a/\mathcal{J}^\perp)^2\right)$ and where p is the wave-vector (or momentum); and b) domain walls, which are boundaries between spin-up and spin-down regions. The domain walls can propagate through $\mathcal{H}_a$, giving them an overall energy of

$E_{p_1} = \mathcal{J}^z/2 - 2\mathcal{J}^a \cos(2p_1)$, with the wave-vector p_1 (or momentum).

With just an infinitesimal applied field along z, the state with one single domain wall becomes too costly in energy due to the macroscopically large number of spins pointing against the field. Instead, the domain wall states are replaced by cluster states, which are bound states of two domain walls, as sketched in the main text, Figure 1(e). Here, the number of overturned spins is finite, and their Zeeman energy can (for now) be neglected. Combining the energies and momenta of the two domain walls, we write $E = E_1 + E_2$, $p = p_1 + p_2$, and $k = p_1 - p_2$. With these definitions the energy of a spin cluster excitation can be written

$$E_{p,k} = \mathcal{J}^z - 4\mathcal{J}^a \cos(k)\cos(p) \tag{9}$$

where $p \in [0, 2\pi]$ is the overall momentum of the spin cluster excitation and k is an internal index, the relative momentum of the two domain walls, which takes values in $[0, \pi]$ for clusters with both an even and odd number of overturned spins.

Inelastic neutron scattering can not probe the transition between the ground state and a cluster state, due to the neutron selection rule $\Delta S = 0, \pm 1$, which allows only to go from the ground state to a spin wave state. However, neutrons can scatter a thermally occupied cluster (spin wave) state into another cluster (spin wave) state.

The matrix element for the scattering of a neutron with momentum transfer q off a cluster state (p, k) into another cluster state (p', k') is maximal when $p' = p + q$ and for the four cases $k' = \pm k \pm q$. This holds for both transverse(S^{xx}) and longitudinal(S^{zz}) scattering. The energy change for the first of these processes is

$$\Delta E = E_{p+q;k+q} - E_{p;k} = 4\mathcal{J}^a \sin(q)\sin(q + p + k). \tag{10}$$

This energy change for a fixed q is maximal when $\sin(q + p + k) = 1$, thus the range of energy transfers is restricted to $\Delta E \le |4\mathcal{J}_a \sin(q)|$. Taking the derivative of ΔE with respect to u we get:

$$\begin{aligned} \left|\frac{\partial \Delta E}{\partial u}\right| &= |4\mathcal{J}^a \sin(q)\cos(q + u)| \\ &= \sqrt{(4\mathcal{J}^a \sin(q))^2 - (\Delta E)^2}, \end{aligned} \tag{11}$$

which implies that the density of scattering states will contain the factor:

$$g(\omega) \propto \frac{1}{\sqrt{(4\mathcal{J}^a \sin(q))^2 - \omega^2}} \tag{12}$$

that diverges as an inverse square root at the upper boundary where $\omega = |4\mathcal{J}^a \sin(q)|$ for $q \neq 0, \pi$. For $q = 0$ or $q = \pi$ the divergence is more dramatic as it then goes as $1/\omega$. The other three scattering processes give the same result for the density of states. The effects due to the thermal occupation can be estimated using the fact that the biggest contributions come from the lowest lying occupied states which have $p = 0$ and $k = 0$ and $p = \pi, k = \pi$. Neutrons with momentum transfer q will scatter from these states with an energy transfer:

$$\Delta E = 4\mathcal{J}^a \sin^2(q), \tag{13}$$

which means that the thermal occupation favours scattering into states with a slightly lower energy transfer than the maximum $4\mathcal{J}^a \sin(q)$. This difference is again largest close to $q = 0$ and $q = \pi$. This is the ferromagnetic equivalent of the Villain mode in an antiferromagnetic Ising chains [10], since a ferromagnetic Ising chain can be mapped onto its antiferromagnetic counterpart by rotating every second spin 180° around the x-axis and replacing $\mathcal{J}^a$ with $\mathcal{J}^\perp$. The antiferromagnetic Villain mode has been observed experimentally e.g. in the Co-based systems $CsCoCl_3$ and $CsCoBr_3$ [11, 12].

Neutrons with momentum transfer q can also scatter from a spin wave with momentum p into another spin wave with momentum $p + q$. Such a process only yields non-zero contributions to the longitudinal scattering channel, $S^{zz}(q, \omega)$, and leads to an energy difference

$$\Delta E = E_{\mathrm{sw,p+q}} - E_{\mathrm{sw,p}} = 4\mathcal{J}^s \sin(q/2)\sin(q/2 + p) \tag{14}$$

which implies that the possible neutron energy transfers are restricted to $\Delta E \le |4\mathcal{J}^s \sin(q/2)|$. A similar computation to the one for cluster states gives a scattering density of states

$$g_{\mathrm{sw}}(\omega) \propto \frac{1}{\sqrt{(4\mathcal{J}^a \sin(q/2))^2 - \omega^2}} \tag{15}$$

which diverges near the upper boundary $\omega = |4\mathcal{J}^a \sin(q/2)|$.

In a longitudinal magnetic field the spin wave dispersion is lifted a constant amount proportional to the field, and for strong enough fields $\mathcal{J}^s \to \mathcal{J}^\perp$. Thus the only change to $g_{\mathrm{sw}}(\omega)$ in a magnetic field is the reduction of $\mathcal{J}^s \to \mathcal{J}^\perp$.

SUPPLEMENTARY NOTE 7: ADDITIONAL DETAILS ON THE INELASTIC NEUTRON SCATTERING EXPERIMENT

The principle behind our inelastic neutron scattering experiment is displayed in Supplementary Figure 5, The initial energy E_i and initial wave vector k_i of the neutron is selected with a monochromator crystal by Bragg scattering by the angle $2\theta_M$, then the sample scatters the beam by the angle $2\theta_S$ and finally the analyser determines the final energy E_f and k_f by Bragg scattering by an angle $2\theta_A$, in the same way as the monochromator. The inelastic neutron scattering measurements were carried out at the MACS cold-neutron spectrometer at the NIST Center for Neutron Research [14] and at the cold

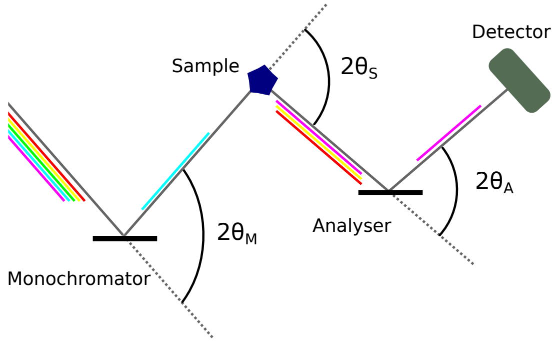

FIG. 5. A schematic drawing of an inelastic neutron scattering experiment using a 3-axis spectrometer, showing the three scattering angles, $2\theta_M$, $2\theta_S$ and $2\theta_A$.

triple-axis spectrometer ThALES at the Institut Laue-Langevin [15]. For both experiments, the sample consisted of two single crystals of $CoCl_2 \cdot 2\,D_2O$ that were mounted on an aluminum sample holder and aligned in the (H,0,L) plane. This sample was mounted in a vertical cryomagnet allowing for longitudinal magnetic fields along the (0,K,0)-direction.

The layout of the MACS instrument, while being based on this principle, is more elaborate. The scattered beam was analysed by a 20 channel detection system equipped with double-bounce pyrolytic graphite analyser crystals [14] with a fixed final energy of $E_f = 3.0$ meV. A Be filter was placed before the monochromator and a BeO filter after the sample. The maximum energy transfer was determined by the difference between the final energy of 3.0 meV and the upper energy cutoff at 5.2 meV of the Be-filter in the incident beam. The sample orientation with respect to the incident beam was fixed in all measurements. For each energy transfer, $S(Q,\omega)$ was probed for several orientations of the detection system. In Supplementary Figure 6 the detector trajectories in the (H,0,L) plane is shown for selected energy transfers. The background contribution from the empty cryomagnet was measured using the same configuration and subtracted from the signal obtained with the sample.

The additional measurements at the ThALES spectrometer were carried out using a pyrolytic graphite monochromator and a pyrolytic graphite analyser. The final energy of the spectrometer was kept fixed at 4.98 meV ($k_f = 1.55$ Å^{-1}). A velocity selector was used in the incoming beam as a filter for second order neutrons of the monochromator and a Be-filter was placed in-between the sample and the analyser.

In the data analysis, an effective background model was used to describe both the incoherent scattering and a field-dependent contribution from the edge of the spin wave continuum, shown in Figure 5 of the main text, using the expression:

$$I_{BG} = A_1 * \exp(-\hbar\omega/A_2) + C, \qquad (16)$$

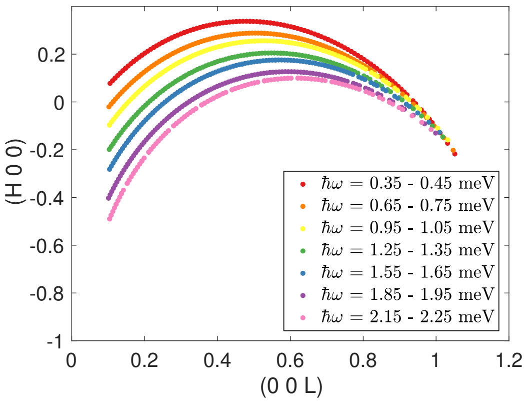

FIG. 6. The MACS detector trajectories for our experimental configuration: $a_3 = 45°$ and $E_f = 3.0$ meV for selected energy transfers.

where the constants A_2 and C were assumed to be the same for all scans. In Supplementary Table IV the parameters for different values of magnetic field are listed.

TABLE IV. Parameters of the effective background for both the MACS and ThALES data.

instrument	$\mu_0 H$ (T)	A_1 (a.u.)	A_2 (a.u.)	C (a.u.)
MACS	6.0	147 ± 5	0.20	3.46
MACS	7.0	138 ± 5	0.20	3.46
MACS	8.0	122 ± 5	0.20	3.46
MACS	9.0	124 ± 5	0.20	3.46
ThALES	7.0	1120 ± 40	0.20	26.0
ThALES	8.0	1150 ± 40	0.20	26.0
ThALES	9.0	1190 ± 40	0.20	26.0
ThALES	10.0	1220 ± 120	0.20	26.0
ThALES	11.0	1170 ± 120	0.20	26.0
ThALES	12.0	1290 ± 40	0.20	26.0
ThALES	13.5	1290 ± 120	0.20	26.0

SUPPLEMENTARY NOTE 8: DATA FROM THE NEUTRON EXPERIMENTS

As a supplement to the MACS data shown in Figure 2 of the main text, an inelastic neutron scattering intensity map as a function of energy and momentum transfer is shown in Supplementary Figure 7 for a magnetic field of 6 T.

The additional excitation spectra at $Q = (0,0,1)$ is shown in Supplementary Figs. 8 and 9 for the different values of magnetic fields. The results of the fits to each of these scans are shown in Figure 6 of the main text.

Supplementary Table V summaries the fitted position ($\hbar\omega_B^*$), amplitude and width (FWHM) of the MBO peak.

The second excitation seen in Supplementary Figure 9(a),(b),(e) and (f) around 4.5 meV, can be ascribed

to a spin wave excitation of the ferromagnetic chains. In Supplementary Figure 10 the intensity of the ferromagnetic spin wave excitation is shown at temperatures 1.5 K and 22 K. A significant softening of the spin wave mode is seen as the temperature is raised above T_N at zero field [17], a change in FWHM from 0.32 meV to 1.19 meV and at 22 K the excitation can be interpreted as a paramagnon. It seems evident that a thermal broadening of the spin wave mode would also imply a broadening of the MBO-excitations.

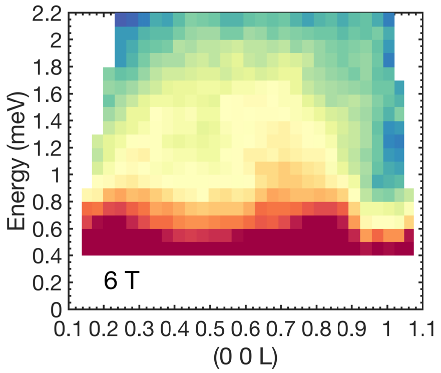

FIG. 7. Inelastic neutron scattering intensity. Data collected at 22 K and 6 T at the MACS spectrometer.

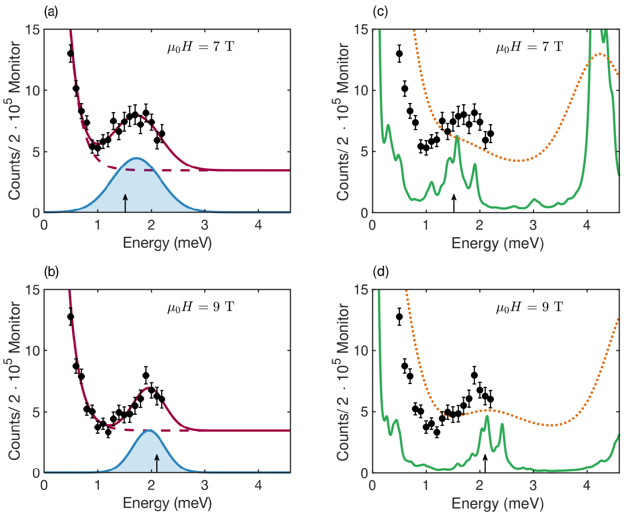

FIG. 8. (a)-(d) Neutron scattering intensity for magnetic fields of 7 T and 9 T, respectively and at 22 K measured at MACS, averaged over the momentum range L = 0.9 − 1.0 r.l.u. Panels (a)-(b) show excitation spectra together with the fit to the data (solid red lines) and background (dashed red lines) described in the main text and the shaded blue area to their difference - the MBO signal. Black arrows are the predicted effective Bloch energies, $\hbar\omega_B^*$, given by Eq. (7) of the main text. Panels (c)-(d) show excitation spectra together with the RPA calculations of the spectra (solid green line) and the RPA calculations with a Gaussian convolution (dashed orange line) as described in the main text.

Supplementary Figure 11 shows the intensity of the MBO signal obtained from the fits shown in Figure 5 of the main text. The signal intensity is predicted to be proportional to $\frac{1}{1-\exp(-\hbar\omega_B^*/k_B T)}$.

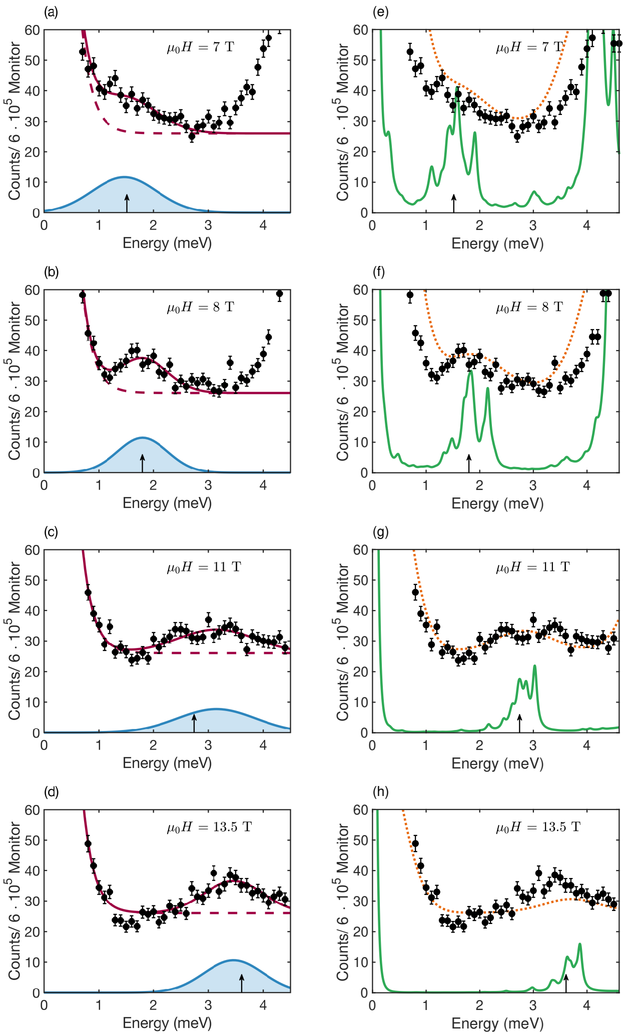

FIG. 9. (a)-(h) Neutron scattering intensity for magnetic fields of 7 T, 8 T, 11 T and 13.5 T, respectively and at 22 K measured at ThALES [16]. Panels (a)-(d) show excitation spectra together with the fit to the data (solid red lines) and background (dashed red lines) described in the main text and the shaded blue area to their difference - the MBO signal. Black arrows are the predicted effective Bloch energies, $\hbar\omega_B^*$, given by Eq. (7) of the main text. Panels (e)-(h) show excitation spectra together with the RPA calculations of the spectra (solid green line) and the RPA calculations with a Gaussian convolution (dashed orange line) as described in the main text. In panels (a),(b),(e) and (f) the paramagnon excitation is visible above 3 meV.

TABLE V. The fitted parameters of the MBO signal.

instrument	$\mu_0 H$ (T)	$\hbar\omega_B^*$ (meV)	Amp (a.u.)	FWHM (meV)
MACS	6.0	1.56 ± 0.04	4.4 ± 0.4	1.11 ± 0.11
MACS	7.0	1.72 ± 0.05	4.5 ± 0.4	1.06 ± 0.12
MACS	8.0	1.77 ± 0.03	5.1 ± 0.4	0.78 ± 0.08
MACS	9.0	1.96 ± 0.06	3.5 ± 0.4	0.71 ± 0.14
ThALES	7.0	1.47 ± 0.07	11.6 ± 0.9	1.40 ± 0.17
ThALES	8.0	1.80 ± 0.04	11.4 ± 0.9	1.08 ± 0.10
ThALES	9.0	2.15 ± 0.05	9.8 ± 0.9	1.13 ± 0.12
ThALES	10.0	2.65 ± 0.06	9.6 ± 0.8	1.39 ± 0.14
ThALES	11.0	3.15 ± 0.08	7.8 ± 0.8	1.68 ± 0.19
ThALES	12.0	3.23 ± 0.06	11.2 ± 0.9	1.20 ± 0.11
ThALES	13.5	3.46 ± 0.05	10.6 ± 0.9	1.27 ± 0.12

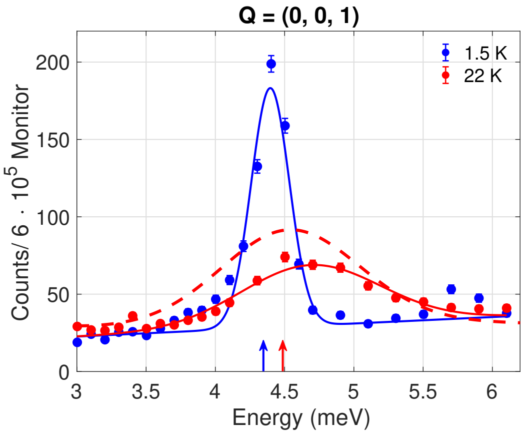

FIG. 10. Neutron scattering intensity at the temperatures 1.5 K (blue circles) and 22 K (red circles) and at a magnetic field of 8 T. Solid lines are gaussian fits to the peaks. The dashed red line shows the RPA calculations convoluted with a gaussian function using the same parameters as in the main text. The blue and red arrows corresponds to the energy ferromagnetic spin wave excitation as predicted by the RPA calculations at 1.5 K and 22 K, respectively. Data taken at ThALES [16].

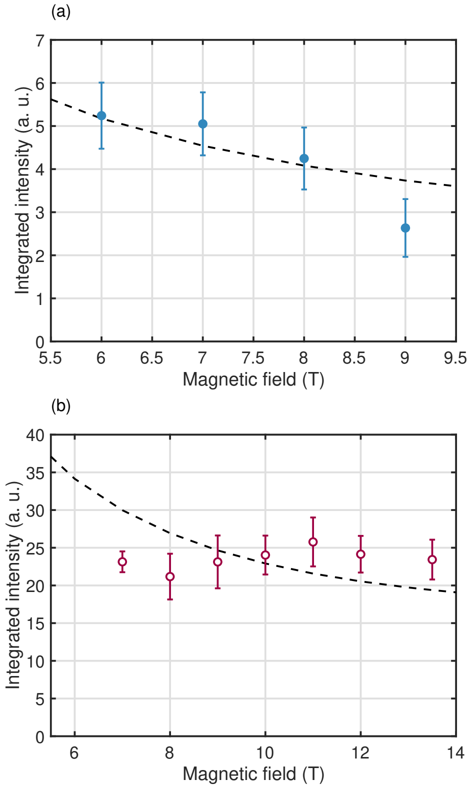

FIG. 11. Field dependence of the intensity of the MBO signal. (a) Results obtained from MACS data are shown with solid blue circles and (b) results obtained from ThALES data are shown with open red circles. The dashed line is a fit to the predicted behaviour, see text for more details.

SUPPLEMENTARY REFERENCES

[1] Morosin, B. and Graeber, E. J. A reinvestigation of the crystal structure of $CoCl_2 \cdot 2\,H_2O$, *Acta Cryst*, 16, 1963.

[2] Cox, D. E., Frazer, B. C. and Shirane, G. The magnetic structure of $CoCl_2 \cdot 2\,D_2O$. *Physics Letters*, 17(2):103–104, 1965.

[3] Abragam, A. and Pryce, M. H. L. The Theory of Paramagnetic Resonance in Hydrated Cobalt Salts. Proceedings of the Royal Society A: Mathematical, Physical and Engineering Sciences, 206(1085):173–191, 1951.

[4] Jensen, J., Larsen, J. and Hansen, U. B. Comprehensive cluster-theory analysis of the magnetic structures and excitations in $CoCl_2 \cdot 2\,H_2O$. *Physical Review B*, 97(2):024423, 2018.

[5] Narath, A. Antiferromagnetism in $CoCl_2 \cdot 2\,H_2O$. 1. Magnetic Structure. *Physical Review*, 136(3A):A766–A771, 1964.

[6] Tinkham, M. Microscopic Dynamics of Metamagnetic Transitions in an Approximately Ising System: $CoCl_2 \cdot 2\,H_2O$. *Physical Review*, 188(2):967–973, 1969.

[7] Narath, A. Antiferromagnetism in $CoCl_2 \cdot 2\,H_2O$. 2. Chlorine Nuclear Magnetic Resonance and Paramagnetic Susceptibility. *Physical Review*, 140(2A):A552–A568, 1965.

[8] E. Prince, editor. *International Tables for Crystallography - Vol. C - Chapter 4 - Magnetic formfactors*, volume C of *International Tables for Crystallography*. International Union of Crystallography, Chester, England, 2006.

[9] Shinkevich, S. and Syljuåsen, O. F. Spectral signatures of magnetic Bloch oscillations in one-dimensional easy-axis ferromagnets, *Physical Review B*, 85:104408, 2012.

[10] Villain, J. Propagative spin relaxation in the Ising-like antiferromagnetic linear chain, *Physica B+C*, 79(1):1–12, 1975.

[11] Yoshizawa, H., Hirakawa, K., Satija, S. K. and Shirane, G. Dynamical correlation functions in a one-dimensional Ising-like antiferromagnetic $CsCoCl_3$: A neutron scattering study. *Physical Review B*, 23(5):2298–2307, 1981.

[12] Nagler, S. E., Buyers, W. J.L., Armstrong, R. L. and Briat, B. Propagating domain walls in $CsCoBr_3$. *Physical Review Letters*, 49(8):590–592, 1982.

[13] Momma, K. and Izumi, F. VESTA 3 for three-dimensional visualization of crystal, volumetric and morphology data. *J. Appl. Crystallogr.*, 44, 1272-1276 (2011).

[14] Rodriguez, J. A., Adler, D. M., Brand, P. C., Broholm, C., Cook, J. C., Brocker, C., Hammond, R., Huang, Z., Hundertmark, P., Lynn, J. W., Maliszewskyj, N. C., Moyer, J., Orndorff, J., Pierce, D., Pike, T. D., Scharfstein, G., Smee, S. A. and Vilaseca, R. MACS - a new high intensity cold neutron spectrometer at NIST, *Measurement Science and Technology*, 19(3):034023, 2008.

[15] Boehm, M., Steffens, P., Kulda, J., Klicpera, M., Roux, S., Courtois, P., Svoboda, P., Saroun, J. and Sechovsky, V. , ThALES - Three Axis Low Energy Spectroscopy for highly correlated electron systems, *Neutron News*, 26:3, 18-21, 2015

[16] Hansen, U. B., Boehm, M., Christensen, N. B. and Lefmann, K., Magnetic Bloch Oscillations and domain wall dynamics in a near-Ising ferromagnetic chain, *Institut Laue-Langevin (ILL)*, doi:10.5291/ILL-DATA.4-04-500

[17] Kjems, K. J., Als-Nielsen, J. and Fogedby., H. Spin-wave dispersion in $CoCl_2 \cdot 2\,D_2O$: A system of weakly coupled Ising chains, *Physical Review B*, 12(11), 5190-5197, 1975.
